# Polymorphism of the *FTO* Gene Influences Body Weight in Children with Type 1 Diabetes without Severe Obesity

**DOI:** 10.1155/2014/630712

**Published:** 2014-08-19

**Authors:** Włodzimierz Łuczyński, Wojciech Fendler, Anna Ramatowska, Agnieszka Szypowska, Agnieszka Szadkowska, Wojciech Młynarski, Miron Chumiecki, Przemysława Jarosz-Chobot, Joanna Chrzanowska, Anna Noczyńska, Agnieszka Brandt, Małgorzata Myśliwiec, Barbara Głowińska-Olszewska, Paweł Bernatowicz, Oksana Kowalczuk, Artur Bossowski

**Affiliations:** ^1^Department of Pediatrics, Endocrinology, Diabetology with Cardiology Division, Medical University of Bialystok, Bialystok 15-274, Poland; ^2^Department of Paediatrics, Oncology, Haematology and Diabetology, Medical University of Lodz, Lodz 91-738, Poland; ^3^Department of Paediatrics, Medical University of Warsaw, Warsaw 01-184, Poland; ^4^Department of Paediatrics, Paediatric Endocrinology and Diabetology, Medical University of Silesia, in Katowice, Katowice 40-752, Poland; ^5^Department of Endocrinology and Diabetology of Children and Adolescents, Wroclaw Medical University, Wroclaw 50-268, Poland; ^6^Department of Paediatrics, Diabetology and Endocrinology, Medical University of Gdansk, Gdansk 80-211, Poland; ^7^Department of Haematology, Medical University of Bialystok, Bialystok 15-274, Poland; ^8^Department of Clinical Molecular Biology, Medical University of Bialystok, Bialystok 15-274, Poland

## Abstract

The objective was to compare the impact of clinical and genetic factors on body mass index (BMI) in children with type 1 diabetes (T1DM) without severe obesity. A total of 1,119 children with T1DM (aged 4–18 years) were qualified to take part in the study. All children were genotyped for variants of *FTO*,* MC4R*,* INSIG2*,* FASN*,* NPC1*,* PTER*,* SIRT1*,* MAF*,* IRT1*, and* CD36*. *Results.* Variants of* FTO* showed significant association with BMI-SDS in the T1DM group. The main factors influencing BMI-SDS in children with T1DM included female gender (*P* = 0.0003), poor metabolic control (*P* = 0.0001), and carriage of the A allele of the* FTO* rs9939609 gene (*P* = 0.02). *Conclusion.* Our research indicates, when assessing, the risk of overweight and obesity carriage of the A allele in the rs9939609 site of the* FTO* gene adds to that of female gender and poor metabolic control. This trial is registered with ClinicalTrials.gov (NCT01279161).

## 1. Introduction

The incidence of overweight and obesity among children has greatly increased in the past few decades [1]. Our stable genetic system, favouring the accumulation of energy, has become a problem in times of excessive access to food and low physical activity. Recently, promising data has been found regarding polymorphisms of* FTO*,* MC4R*,* INSIG2*,* CD36* genes, and many others [2]. The influence of FTO and MC4R genes on body mass is mediated primarily through food consumption (appetite regulation) rather than through a decrease in activity-related energy expenditure (review [3]). Controversial association results for INSIG2 on body mass index may be explained by interactions with age and with MC4R [4].

The intensive insulin therapy for type 1 diabetes (T1DM) is associated with weight gain, abdominal obesity, dyslipidaemia, hypertension, and the presence of features of atherosclerosis development in imaging [5]. For unknown reasons, sex differences are observed in this field: in contrast to the nondiabetic population, girls and women with diabetes are characterized by a greater number of risk factors for cardiovascular diseases than boys and young men [6]. Since the association of known genetic polymorphisms with body weight is not very strong, the objective of this study was to compare the impact of clinical and genetic factors on body mass index in a large cohort of not severely obese children with T1DM.

## 2. Materials and Methods

### 2.1. Patients and Methods

A total of 1,119 children with T1DM from study centers of the PolPeDiab research group were qualified to take part in the study. These six centers have treated 60% of children with diabetes in Poland (10–90% of patients from each center). The study inclusion criteria were Polish ethnicity, age 4–18 years, diagnosis of T1DM (according to ISPAD), treatment with insulin alone for at least one year using the same intensive regimen with the use of pens or personal insulin pumps, and end of remission (based on the definition suggested by Mortensen et al.: HbA1c% + (4 × insulin dose U/kg/day) > 9 [7]). Psychiatric abnormalities (including nutritional disorders), evidence of chromosomal disorders in physical examination, and hormonal and autoimmune disorders (celiac disease, diseases of the thyroid, adrenals, and gonads) were excluded (20%). Children with severe obesity (SDS-BMI > 3) were also excluded from the study (4% of all patients with T1DM). Subjects did not take any medications apart from insulin. The study was conducted between October 2009 and December 2011. The study design was approved by the Ethics Committee at the Medical University of Bialystok (no. R-I-002/347/2009). The parents/guardians of each subject gave their written, informed consent for study participation.

### 2.2. Anthropometric Methods, Additional Assessments, and Definitions

Subject examination included measurements of height (cm, Harpenter's stage-meter) and weight (kg) conducted by trained members of the study team. Height and weight were measured in light sports garments or underwear, without shoes, with an empty bladder. Each measurement was taken twice. Height was measured with a precision of 0.1 cm. With the difference in height measurements of >0.5 cm a third measurement was taken. Weight was measured with a precision of 0.1 kg; with the results divergent by >0.3 kg, a third measurement was taken. The BMI (kg/m^2^) and BMI-SDS values were calculated [8]. The intra- and interobserver agreements were calculated according to the statistical methods proposed by Bland and Altman [9]. The intra- and interobserver coefficient of variation was 1.0% and 1.5% (resp.) for the height and 0.8% and 1.1% for weight. The collected data were referenced against the recently updated norms for Polish children, including weight centile charts according to sex and age (Polish nationwide OLAF project) [8].

The daily insulin dosage was acknowledged to be the mean dosage from the 3 days prior to the current visit (in the case of pens, the data was obtained from blood glucose self-monitoring diaries, and in the case of insulin pumps the data was obtained from device software). Study subjects were on a diabetic diet, they calculated carbohydrate and protein-fat equivalents (pumps), and all had been educated on functional insulin therapy.

Glycosylated hemoglobin (HbA1c) rates were evaluated with the use of high-performance liquid chromatography (HPLC) in the reference laboratories. HbA1c values obtained from the laboratories were mathematically standardized to the Diabetes Control and Complications Trial normal range. Mean HbA1c for the previous year of treatment (minimum 3 measurements) were calculated. Only children with all data available were qualified for analysis.

### 2.3. Genotyping

All children were assessed for the following polymorphisms:* FTO* (rs9939609: A/T, rs1421085: C/T, rs17817449: G/T, rs1121980: A/G),* MC4R* (rs17782313: C/T),* INSIG2* (rs7566605: C/G),* FASN* (rs2229422: A/G),* NPC1* (rs1805081: C/T),* PTER* (rs10508503: C/T),* SIRT1* (rs7895833: A/G, rs1467568: A/G),* MAF* (rs1424233: C/T), and* CD36* (rs3211867: A/C, rs3211883: A/T, rs1527483: A/G) via allelic discrimination with ABI 7900HT Fast Real-Time PCR System with SDS 2.1 software (Applied Biosystems, Foster City, CA, USA). During blood sampling for required laboratory diagnostic tests, a volume of 0.5 mL of blood was collected for genotyping. All children were assessed for the polymorphisms via allelic discrimination with ABI 7900HT Fast Real-Time PCR System with SDS 2.1 software (Applied Biosystems, Foster City, CA, USA). The genetic variants were evaluated with the use of validated commercially available probes included in the TaqMan SNP Genotyping Assays (Applied Biosystem). DNA was isolated from venous whole blood with TaqMan Sample-to-SNP Kit (Applied Biosystems) according to the manufacturer's instructions. The assay was conducted on 384-well plates in 5 *μ*L; the mixture contained 1 *μ*L of DNA, 2.5 *μ*L TaqMan GTXpress Master Mix (Applied Biosystems), 0.25 *μ*L TaqMan Genotyping Assay Mix 20x (Applied Biosystems), and 1.25 *μ*L DNase-free water. The temperature profile of the reaction was as follows: initial denaturation at 95°C for 10 min, 40 consecutive cycles of denaturation at 92°C for 15 s, and hybridization/elongation at 60°C for 60 s. As contamination control, each reaction plate contained negative assays containing water instead of DNA. A total of 99.3% correct reactions were recorded.

### 2.4. Statistical Analysis

Univariate analysis was performed using the Student's *t*-test in the case of continuous variables and the Chi-square test for nominal ones. Frequencies of observed alleles were tested against the Hardy-Weinberg equilibrium using the Chi-square test. As the study was not aimed at establishing individual effects of polymorphisms on the risk of diabetes, we did not perform multivariate association analysis. Multivariate linear regression was used to evaluate the impact of all polymorphic variants and clinical features on BMI-SDS. Second-order interactions were also analysed to evaluate any potential interactions of the presence of diabetes and genotypes at particular loci. The study was planned to provide 80% statistical power to detect differences greater than 0.2 of BMI-SDS with type 1 error probability lower than 0.15, for heterozygosity of the tested SNPs equal to 0.5. Since there were 9 SNPs in question, actual power for respective comparisons ranged between 70 and 90%.

## 3. Results

### 3.1. Study Group Characteristics

Baseline characteristics of the studied group are presented in Table 1. In the group of children with type 1 diabetes, the girls were characterized by higher values of standardized BMI compared to boys (0.52 ± 0.94 versus 0.31 ± 0.91, *P* = 0.0001). In terms of other parameters, there were no gender differences. BMI-SDS did not correlate strongly or significantly with age, duration of diabetes, insulin dose, or HbA1c levels (*r* from −0.007 to 0.093, all *P* values >0.05).

### 3.2. Results of the Genetic Studies

The distribution of alleles at two of the studied loci (*FASN* and* MAF*) deviated from the ones expected in the Hardy-Weinberg equilibrium, which excluded them from further analysis of polymorphic allele carriage effects and multivariate models.

**Figure 1:**
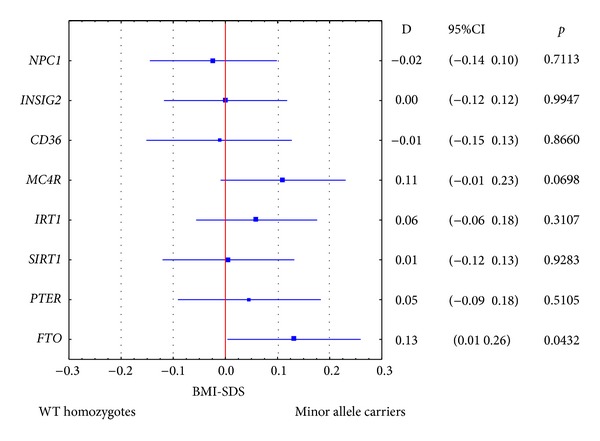
The influence of tested polymorphic variants on standardized BMI (BMI-SDS) in children with type 1 diabetes. D: difference of means, 95% CI: 95% confidence interval, and WT: wild-type.

Of all tested SNPs only* FTO* showed any significant association with BMI-SDS in the T1DM group (Figure 1). Recessive models of SNP effects were tested, but, apart from* FTO*, which was significant for both recessive and dominant modes of action, none of the analysed polymorphisms showed differences even of borderline significance (*P* > 0.1 in all cases).

Carriers of* FTO* polymorphic allele had shown higher BMI-SDS scores regardless of the patient's gender (Table 2). The impact of the patient's age and other genetic factors remained nonsignificant (*P* > 0.15). Except for female gender and carriage of the polymorphic allele of* FTO*, the third significant factor associated with higher BMI-SDS was confirmed to be HbA1c level (Table 2).

## 4. Discussion

Our study of the group of individuals without severe obesity indicates an effect of* FTO* gene polymorphism on BMI in children with T1DM. Moreover, gender and metabolic control significantly influenced standardized BMI.

Previously we have shown an association of* FTO* with body weight in Polish healthy children, similar to other authors [10, 11]. Nevertheless, it seems that there is no correlation between the genotype predisposing to obesity and the risk of autoimmune reactions and the formation of T1DM [12]. On the other hand, overweight did not predispose to more rapid progression of autoimmune response in patients enrolled in the study BABYDIAB [13]. According to the data of other authors, the influence of the genetic polymorphisms we studied on body weight is not strong, and severe obesity is due to other causes. Therefore, we excluded from the current analysis children with a value of standardized BMI above 3. However, our research indicates, when assessing, the risk of overweight and obesity carriage of the A allele in the* FTO* variant rs9939609 should be taken into account. The impact of this polymorphism on body weight in patients with T1DM was also observed in a study of the genetics of kidney diseases in diabetes [14]. The most interesting result of our observations is the fact that* FTO* variants have an additional effect, besides clinical features, on the body weight in diabetic children.

The* FTO, INSIG2, CD36,* and* MC4R* genes tested in our study are the main polymorphisms associated with body weight [15]. Substantial problems concerning obesity and T1DM include difficulty in distinguishing the types of disease in adolescents with features of insulin resistance, weight gain during intensive insulin therapy, the potential impact of obesity on long-term complications, and the relationship of body weight with metabolic control. In a large group of German and Austrian children with type 1 diabetes a higher incidence of risk factors for cardiovascular disease in girls compared to boys (metabolic control, body mass index, total cholesterol, triglycerides, and blood pressure) was observed, except for abnormal HDL-cholesterol and cigarette smoking [16]. The reasons for poor metabolic control and obesity in adolescent girls with type 1 diabetes remain unknown. Obviously, patients with obesity, poor self-control, and poor metabolic control constitute a risk group for eating disorders [17].

Recently, a significant association between the body weight of adolescents with type 1 diabetes and their parents' body weight was demonstrated [18]. Interestingly, metabolic control correlated not only with the patients' body weight, but also with that of their parents. It is therefore clear that obesity is a familial problem for children with T1DM. It remains an open question whether, in these cases, obesity has an environmental background (the same style of life) or genetic background.

## 5. **Conclusions**

The main factors influencing body weight in children with diabetes include female gender, poor metabolic control, and carriage of the A allele of the* FTO* rs9939609 variant. Improvement of metabolic control of T1DM is the major modifiable factor associated with BMI and should be considered of crucial importance, particularly in girls T1DM and high BMI.

## Figures and Tables

**Table 1:** Clinical data of children with diabetes.

	T1DM	
	Mean	SD
Age	13.39	3.42
Height-SDS	0.22	1.25
Weight-SDS	0.50	1.06
SDS-BMI	0.42	0.94
HbA1c	7.77	1.52
Insulin dose (U/kg/day)	0.79	0.25

**Table 2:** Multivariate regression model including the clinical and genetic features influencing the standardized body mass index in children with type 1 diabetes.

Factors affecting BMI among children with diabetes	Regression coefficient	Partial correlation coefficient	*P*
Intercept	−0.201654		0.205718
Female sex	0.110525	0.119130	0.000337
FTO polymorphic allele carriage	0.075598	0.074232	0.024979
Mean yearly HbA1c level	0.075028	0.124044	0.000190
